# Valproic acid-induced hyperammonemic encephalopathy (VIHE) in a patient with Bipolar disorder: A case report and literature review

**DOI:** 10.1192/j.eurpsy.2022.880

**Published:** 2022-09-01

**Authors:** E. Isidahome, M. San Gabriel

**Affiliations:** Mount Sinai School of Medicine, Psychiatry, ELMHURST, United States of America

**Keywords:** VIHE - Valproic acid induced hyperammonemic encephalopathy, bipolar disorder, VPA - Valproic acid, Schizoaffective disorder

## Abstract

**Introduction:**

Valproic acid (VPA) is a valuable treatment for bipolar disorder, schizoaffective disorder, and agitation^1^. However, potential side-effects include sedation, headaches, tremors, ataxia, gastrointestinal issues, neural tube defect, ^3^ and mild hyperammonemia even in normal liver function test ^1^ and VPA level.

**Objectives:**

To illustrate clinical presentation of VIHE and provide literature review on post-VIHE treatment options.

**Methods:**

A 59-year-old male with PMH of Diabetes Mellitus, Hypertension, Hyperlipidemia, LVH, COPD, s/p CVA, and PPH of schizoaffective disorder, bipolar type. Patient stable on VPA 1250mg daily and Olanzapine 5mg daily for >2years until recent manic decompensation resulting to up-titration of VPA to 1500mg H.S. Thereafter, he presented with altered mental status, with VPA level (111.4 ug/ml), hyponatremia (119 mmol/L) and hyperammonemia (84 umol/L). Subsequently, admitted as a case of VIHE and hyponatremia.

**Results:**

VPA has shown to cause hyperammonemia alone or when combined with antipsychotics^6^. VIHE reported in up to 47.7% of patients on VPA^1^, but symptomatic in approximately 10% of patients on VPA with blood ammonia level about 2-fold the normal range^8^. VIHE presents with confusion, ataxia, blurred vision, delirium, and seizures^3^. Treatment options include VPA discontinuation, switch to other mood stabilizers (lithium carbonate, lamotrigine), utilization of medications to lower blood ammonia levels (Lactulose, Rifaximin/Neomycin) ,^3^ antipsychotic monotherapy, and supplements (Levocarnitine or Carglumic acid) in the prevention, maintenance, and treatment of VIHE. These supplements can be added to VPA if the benefits of re-initiating or continuing VPA outweighs the risk^3^.

**Conclusions:**

Further research is needed.

**Disclosure:**

No significant relationships.

